# Comprehensive Repertoire of Foldable Regions within Whole Genomes

**DOI:** 10.1371/journal.pcbi.1003280

**Published:** 2013-10-24

**Authors:** Guilhem Faure, Isabelle Callebaut

**Affiliations:** CNRS, UPMC Univ Paris 6, IMPMC, UMR7590 - IUC, Paris, France; Indiana University, United States of America

## Abstract

In order to get a comprehensive repertoire of foldable domains within whole proteomes, including orphan domains, we developed a novel procedure, called SEG-HCA. From only the information of a single amino acid sequence, SEG-HCA automatically delineates segments possessing high densities in hydrophobic clusters, as defined by Hydrophobic Cluster Analysis (HCA). These hydrophobic clusters mainly correspond to regular secondary structures, which together form structured or foldable regions. Genome-wide analyses revealed that SEG-HCA is opposite of disorder predictors, both addressing distinct structural states. Interestingly, there is however an overlap between the two predictions, including small segments of disordered sequences, which undergo coupled folding and binding. SEG-HCA thus gives access to these specific domains, which are generally poorly represented in domain databases. Comparison of the whole set of SEG-HCA predictions with the Conserved Domain Database (CDD) also highlighted a wide proportion of predicted large (length >50 amino acids) segments, which are CDD orphan. These orphan sequences may either correspond to highly divergent members of already known families or belong to new families of domains. Their comprehensive description thus opens new avenues to investigate new functional and/or structural features, which remained so far uncovered. Altogether, the data described here provide new insights into the protein architecture and organization throughout the three kingdoms of life.

## Author Summary

Spontaneous or induced folding into a specific 3D structure is a key property of proteins to perform their biological functions. Folded 3D structures of proteins perform specific functions, including interactions with other proteins. Intrinsically disordered regions also mediate interaction, gaining structure only when bound to a target protein. In both cases, hydrophobicity generally plays a major role in the protein segment “foldability”. Here, we developed an original procedure to identify foldable segments from only the information of a single amino acid sequence and to explore protein structures at a proteomic scale. Our approach goes beyond the simple consideration of mean hydrophobicity, by including the secondary structure information through the use of a two-dimensional transposition of the sequence. The developed procedure, combined with disorder predictors, may facilitate the specific identification of small segments that undergo coupled folding and binding. Combined with the analysis of specific domain databases, it also highlights orphan foldable segments, which remain yet uncharacterized.

## Introduction

Domains are the modular building blocks of proteins and correspond to recurring, fundamental units of both protein structure and evolution. Protein domains may exist alone, but frequently are part of larger, multi-domain proteins [1]. The advent of complete genomes sequences has led to the estimation that 40% of prokaryotic proteins are multidomain, whereas this number increases to about two thirds in eukaryotes [2]. Protein domains are classified into families; several domain families are common to most species, indicating that there is a limited repertoire, which is used to create the large functional space of proteins [3]. Some domain families, considered as “promiscuous”, occur in diverse protein domain architectures (which are defined as the linear orders of the individual domains in multi-domain proteins) and are especially involved in interaction networks [4].

The recognition of domain family membership for uncharacterized proteins is often a first step towards the understanding of their biological roles. Information about protein domains is stored in dedicated databases, in the form of profiles or hidden Markov models (HMMs), which are constructed through sequence similarity searches. These profiles and HMMs can be searched for detecting the domain composition of proteins, starting from their amino acid sequences [5]. By this way, approximately half of the residues of proteomes can be assigned to well-classified domains, such as those stored in the PfamA classification [2]. The percentage of assigned residues increases when less well-characterized domain databases, such as PfamB, are searched. The remaining residues, representing 10–20% of the proteomes and referred to as “orphan” domains, do not match any known domains [2]. These sequences include disordered structures, among which are found linkers between structured domains, but also folded units, which are difficult to characterize, principally due to their small size or their fast evolution relative to an ancestral protein. These can thus not be easily predicted by these sequence similarity-based methods. The prediction of domain boundaries can also be approached through *ab-initio* methods, which don't have such restrictions as they consider solely the protein sequence. These focus on either globular domains or disordered regions and are based on learning models, using a series of proteins for which information on residue properties is known and algorithms such as artificial neural networks and support vector machines (e.g. [6]–[11]). However, the accuracy of domain boundary prediction is often too low for general, practical use. Improvement of the quality of *ab-initio* predictions has been obtained by hybrid methods, adding evolutionary information (e.g. [12], [13]).

**Figure 1:**
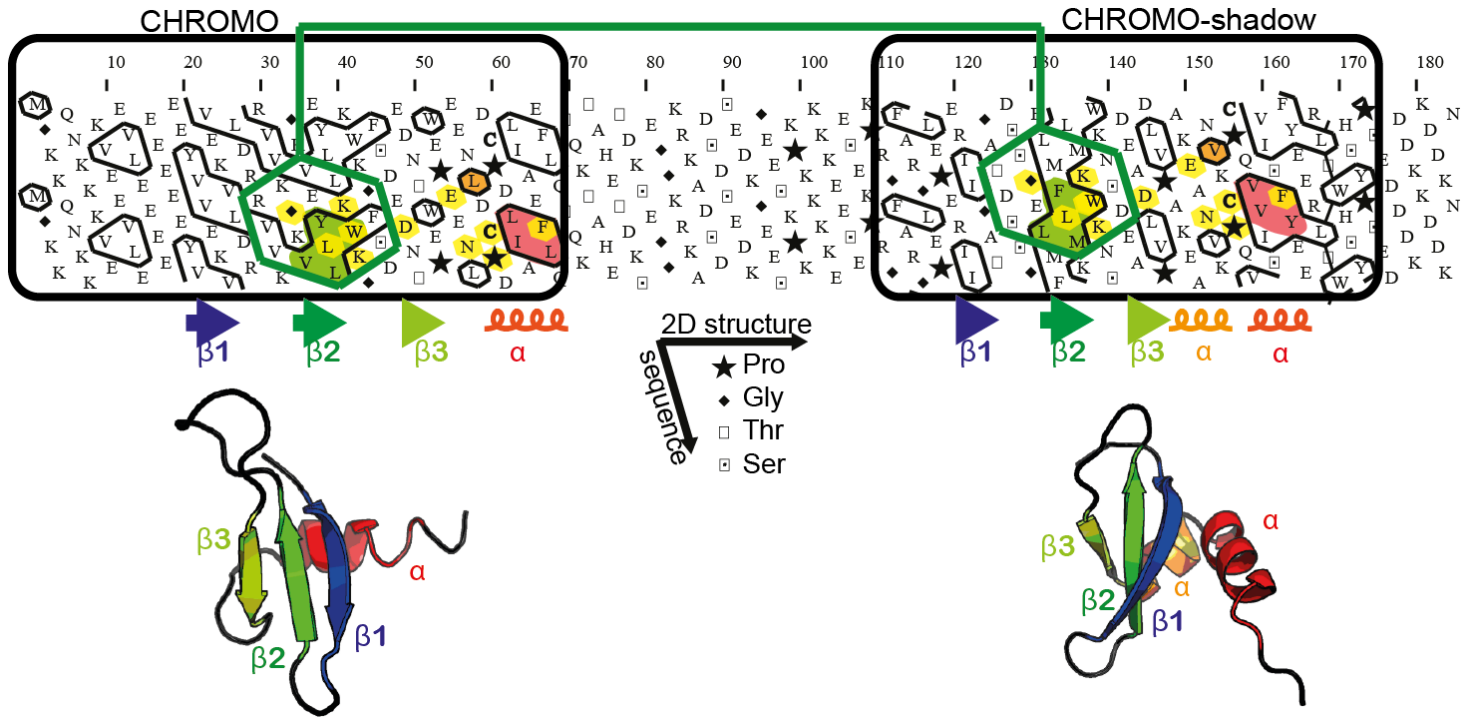
Delineation and comparison of globular domains using HCA. The sequence is written on a duplicated alpha helical net and the hydrophobic amino acids (V, I, L, M, F, W, Y) are contoured [14], [15]. These form hydrophobic clusters, which mainly correspond to regular secondary structures [17]. This is illustrated here with the chromo and chromo-shadow domains of the mouse chromobox homolog 1 (CBX1_MOUSE, UniProt P83917), for which the observed secondary and tertiary structures are shown below the HCA plot (pdb identifiers: 1AP0 and 1DZ1) [66]. Globular domains (boxed), containing approximately one third of hydrophobic amino acids gathered into clusters, are separated by a hinge, which is clearly less hydrophobic. The comparison of the HCA plots of the two domains indicates similar shapes of clusters (shaded green and red), suggesting a structural relationship. This potential relationship is further strengthened by sequence identities (shaded yellow) identified relative to the conserved positions of hydrophobic amino acids within clusters and is supported at the 3D level. The hydrophobic clusters shaded in green and red correspond to the internal strand beta2 and to the C-terminal alpha helix, respectively. In the chromo shadow domain, the loop linking strand beta3 and the C-terminal alpha helix includes a short alpha helix, containing two alanine residues.

Here, in order to get insight into orphan regions corresponding to foldable regions, without consideration of any evolutionary information, we have developed a strategy inspired from our experience in Hydrophobic Cluster Analysis (HCA). This two-dimensional method is used for (i) delineating the position of globular-like domains and (ii) comparing fold signatures at low levels of sequence identity (Fig. 1). HCA is based on the physico-chemical and topological principles underlying the fold of globular domains (dichotomy between hydrophobic/non-hydrophobic amino acids, overall compactness) [14], [15]. It allows a direct statistical access to regular secondary structures gravity centers for a single amino acid sequence through the hydrophobic clusters defined in this way [16], [17]. The immediate information available from this lexical analysis of the protein sequence text is a direct, comprehensive analysis of the protein texture, revealing in particular structured and non-structured regions. Structured regions contain typical hydrophobic clusters, the length of which is similar to those of regular secondary structures, whereas non-structured regions lack or have less and smaller hydrophobic clusters. This property has led to the manual identification of a lot of domain boundaries, constituting crucial starting points for experimental and computational investigations (*see examples at the following url* http://www.impmc.upmc.fr/~callebau/HCA.html). Besides this property, the HCA hydrophobic clusters constitute efficient signatures for comparing remote sequences, allowing to link orphan sequences to known families of domains (*e.g.* [18]) or identify new families of domains (*e.g.* [19]–[21]).

On this basis, we developed a fast and automated procedure, called segmentation-HCA (SEG-HCA) in order to delineate the foldable domains within proteins from the knowledge of their sequences alone and applied it to the characterization of whole proteomes. This approach is distinct from Scooby-Domain [22], [23], which uses the distribution of observed lengths and hydrophobicity in domains with known 3D structures. SEG-HCA also uses a simple binary hydrophobic scale, but enriched from the two-dimensional information highlighting regular secondary structures through the hydrophobic clusters defined by this way. Moreover, SEG-HCA is not limited to the observed lengths in domains with known 3D structures, but cover any foldable region of any length. The information provided by SEG-HCA can then be compared with that included into structural databases, in order to support the structural meaning of the predictions. It can also be compared with that provided by the NCBI's conserved domain database (CDD) [24] in order to highlight «orphan» domains, *i.e.* predicted globular-like domains that don't match any conserved domain (CD). Finally, this information also merits consideration with regard to protein disorder. Intrinsically disordered proteins (IDPs) or Intrinsically unstructured proteins (IUPs) do not, by themselves, assume any stable 3D structures, under physiological conditions [25]–[29]. IUPs however cover different forms of disorder, from totally unfolded chains (“coil”-like or natively unfolded), to coupled folding and binding (*i.e.* disorder-to-order transition), and to pre-molten or molten globules having well-developed secondary structures [29]. We thus also investigated here how eventually a distinction between these different disordered states can be made using the SEG-HCA predictions.

## Results

### SEG-HCA H2CD predictions applied to whole proteomes

**Figure 2:**
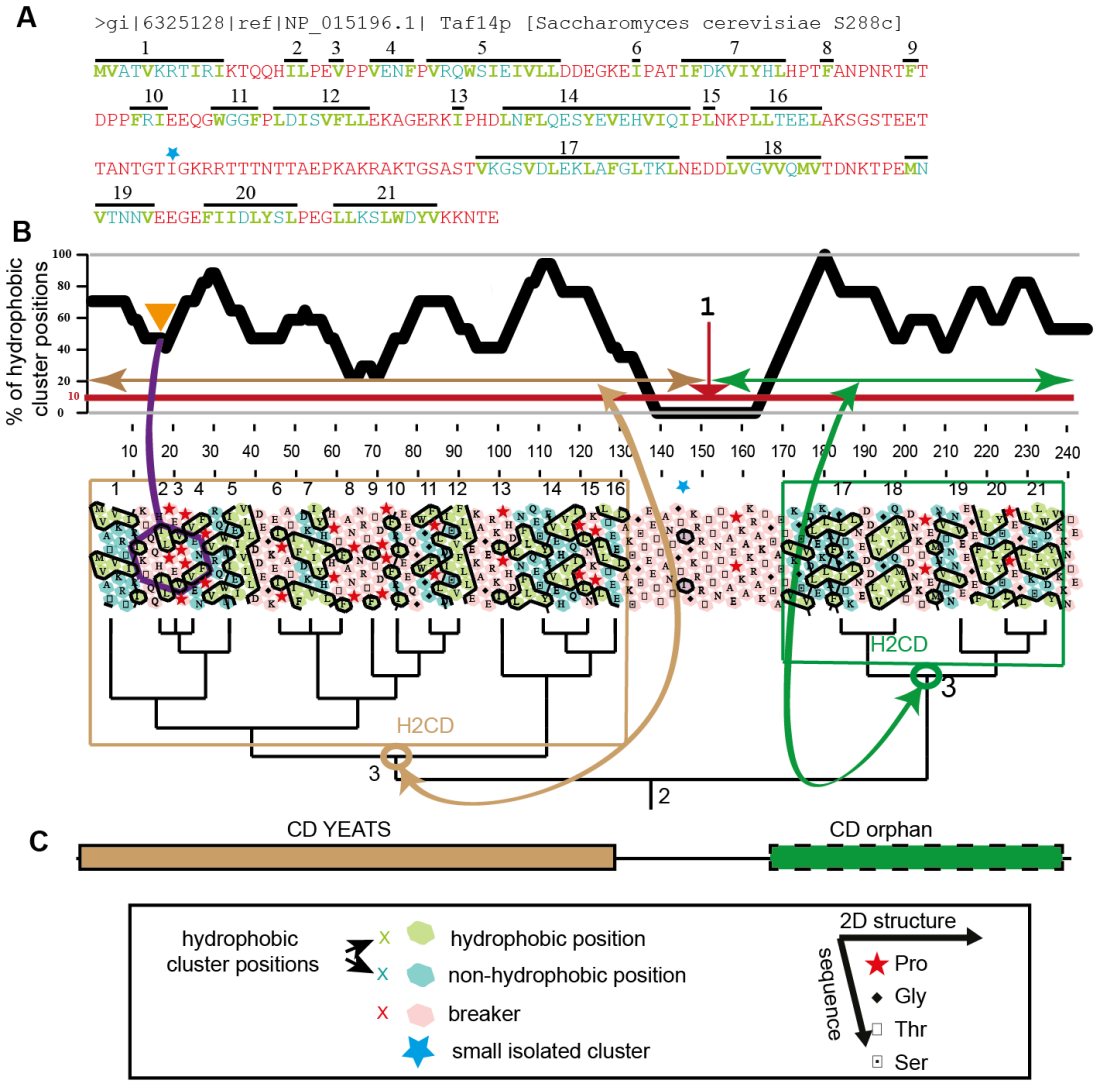
SEG-HCA methodology. (A and B) Hydrophobic amino acids (green) are highlighted in the sequence of *S. cerevisiae* TAF14. At least four consecutive non-hydrophobic amino acids or a proline constitute breakers (red). Hydrophobic amino acids not separated by breaker positions constitute hydrophobic clusters (numbered here 1 to 21), which thus include hydrophobic (green) as well as non-hydrophobic amino acids (green-blue). A single hydrophobic amino acid, which does not contain in its close neighborhood (7 amino acids downstream and upstream) any other hydrophobic cluster is considered as an isolated small cluster and included in the breaker (blue star). The percentage of positions included in hydrophobic clusters (HCP, after hydrophobic cluster position; shaded green and blue) is computed within a sliding window of 17 consecutive residues (one amino acid increment). This interval is represented by the hexagon at the beginning of the sequence, including the second amino acid neighborhood at the HCA 2D level. For the sequence segment included in the hexagon (IKTQQHILPEVPPVENF), the HCP percentage is 47% (8/17). A minimum is identified when this percentage is below 10%. Here, two segments are identified, as a minimum is reached at amino acid 150 (labeled 1). A distance tree is then calculated between hydrophobic clusters. This allows the definition of the limits of regions having high density in hydrophobic clusters (H2CD, after high hydrophobic cluster density), by comparing the different nodes with the segments defined before. Here, the best correspondence is observed between segment 1 and the node including clusters 1 to 16 (labeled 3 brown) and segment 2 and the node including clusters 17 to 21 (labeled 3 green). (C) The predicted H2CD are then compared to the domains assigned by RPS-BLAST from the Conserved Domain Database (CDD). In this example, only the first domain is assigned from CDD (YEATS domain), while the second one remains unassigned by CDD (CD-orphan).

So far, analysis of HCA plots was manual and thus limited to small sets of protein sequences. The SEG-HCA procedure now allows the automation of one aspect of the HCA plot analysis by delineating, from the consideration of a single protein sequence, the positions of segments having a high density in hydrophobic clusters (H2CD segments, Fig. 2). The methodology is fully described in the Material and Methods section. Briefly (Fig. 2), SEG-HCA first defines hydrophobic clusters into a protein sequence, using the current HCA rules, and calculates the percentage of positions included in these hydrophobic clusters (HCP, after Hydrophobic Cluster Positions, shaded green and blue on Fig. 2B), within a sliding window of 17 amino acids (1-amino acid increment). Note that this is not equivalent to a simple calculation of mean hydrophobicity, because non-hydrophobic amino acids within clusters (blue on Fig. 2B) are also taken into account. Hence, the HCP percentage approximates, from the consideration of a single sequence, the density in regular secondary structures. Then, a HCP threshold value of 10% is chosen to define potential hinge regions between segments having a high density in hydrophobic clusters (H2CD), whose positions are then refined through the consideration of a sequence based-hydrophobic cluster distance tree (see Material and Methods for the details).

SEG-HCA now gives access to the analysis of whole proteomes. We collected here the H2CD segments (as predicted by SEG-HCA) for the entire, archetypal proteomes of *Homo sapiens, Saccharomyces cerevisiae, Plasmodium falciparum, Escherichia coli* and *Archeoglobus fulgidus*. The number of predicted H2CD, as well as the total number of amino acids predicted in H2CD, are given in Table 1. The proportion of amino acids in H2CD is higher in Bacteria and Archaea than in Eukarya. In the following text, results will be illustrated for the human proteome, except in some special cases where a different behavior is observed for specific species.

**Table 1:** H2CD and CD segments within proteomes.

	*H. sapiens*	*S. cerevisiae*	*P. falciparum*	*E. coli*	*A. fulgidus*
**Sequences**	34521	5907	5337	4128	2420
**Residues**	18395802	2828010	3979079	1251206	655015
**Mean length (aa)**	533	479	746	303	271
**H2CD**					
**Number of H2CD**	137655	18555	22939	6745	3075
**Number of large H2CD (>50 aa)**	72851	10732	15348	5439	2747
**Number of residues in H2CD**	15879870	2550812	3558433	1223971	650868
**Percentage of residues in H2CD**	86.3	90.2	89.4	97.8	99.4
**Mean H2CD length (aa)**	115	137	155	181	212
**Mean percentage of residues in H2CD per sequence**	77% (5.2)	80% (5)	86% (2.8)	90% (3.5)	94% (2.6)
**CD**					
**Number of CD**	69348	7251	5161	5497	2679
**Number of residues in CD**	7359656	1170126	740778	894558	415889
**Pecentage of residues in CD**	40	41	19	71	63
**Mean CD length (aa)**	106	161	144	163	155
**Mean percentage of residues in CD per sequence**	44% (9.3)	45% (11.2)	29% (10.4)	74% (8.6)	62% (13.2)
**Coverage**					
**CD coverage by H2CD (up to 95%)**	74,8%	75,5%	82,1%	75,2%	86,5%
**CD not covered by large H2CD**	3078	253	82	132	33
**CD not covered by any H2CD**	435	7	9	4	0
**Number of CD orphans (>50 aa)**	27316	4126	12459	525	500
**Percentage of CD orphans (>50 aa)**	19.8	22.2	54.3	7.8	16.2
**Number of CD orphans in NR70 (>50 aa)**	16562	3929	12205	499	492
**CD/H2CD architecture of proteins**					
**CD orphan proteins**	5848 (17%)	1188 (20%)	2035(38%)	271(7%)	438(18%)
**H2CD orphan proteins (>50 aa)**	916 (3%)	185 (3%)	49 (1%)	118 (3%)	55 (2%)
**Mono CD proteins**	14859 (43%)	3125 (53%)	2230 (42%)	2722 (66%)	1509 (62%)
**Mono H2CD proteins (50 aa)**	15588 (45%)	2981 (41%)	2270 (43%)	2920 (71%)	2040 (84%)
**Multiple CD proteins**	13814 (40%)	1594 (27%)	1072 (20%)	1135 (27%)	473 (20%)
**Multiple H2CD proteins (50 aa)**	18017 (52%)	2741 (46%)	3011 (56%)	1090 (26%)	325 (14%)

### SEG-HCA H2CD predictions are more than the converse of disorder predictions and may be used for defining categories in disorder

The prediction of structured regions might be considered as the simple converse of disorder predictions. We thus compared the SEG-HCA predictions to the disorder predictions performed by IUPRED [30], [31]. IUPRED is an available well-recognized method, which considers interaction energies for predicting stretches of amino acids that should not contribute to stable structures. According to the D_2_P_2_ database [32], IUPRED provides among the lowest estimation of global percentage disorder.

We achieved this comparison according two different, but parallel routes.

**Figure 3:**
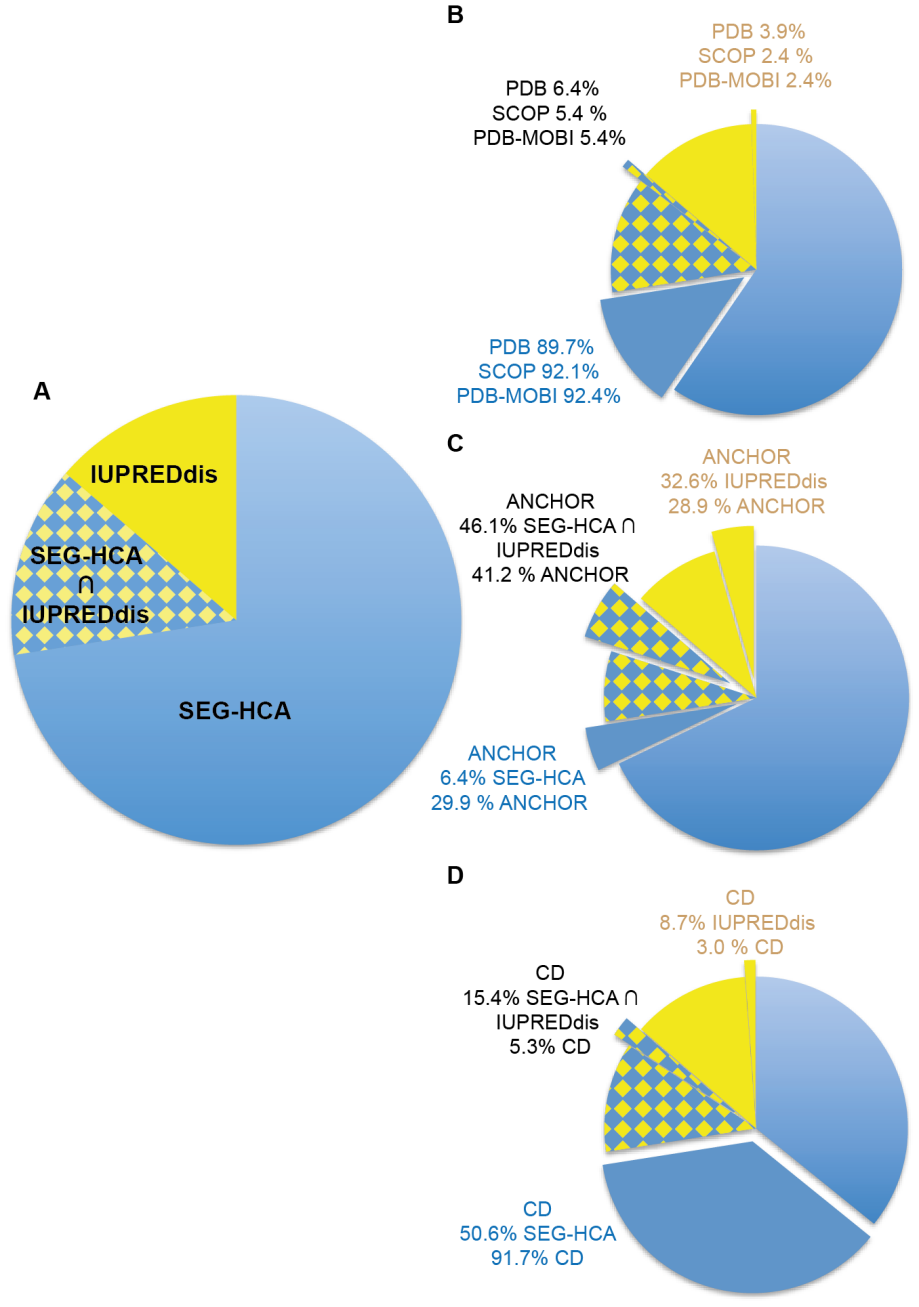
Comparison of SEG-HCA and IUPREDdis predictions. (A) Percentages of amino acids covered by SEG-HCA and IUPRED-L predictions in the human proteome (18395802 residues). Each class (SEG-HCA, IUPREDdis and SEG-HCA∩IUPREDdis) has been searched for coverage by PDB assignments, Mobi-DB-filtered PDB assignments and SCOP assignments (B), as well as ANCHOR predictions (C) and CDD assignments (D).

First, we compared the total number of amino acids predicted in H2CD segments to those predicted as disordered by IUPRED (IUPREDdis). We used for this prediction the long (L) variant of IUPRED, which has been trained on long forms of disorder. These IUPRED-L predictions are in agreement with those reported in the D_2_P_2_ database [32]. According to the current view [32], [33], Eukarya have a higher content in disorder than the two species chosen here for illustrating Bacteria and Archaea (Table 2). However, Bacteria and Archaea show wide disorder distribution, with very low level of predicted disorder for some species, such here observed with the *A. fulgidus* proteome. We show (Fig. 3A and Table 2) that to a large extent, there is a clear relationship between the two predictions, which are opposite. However, SEG-HCA H2CD predictions are not the simple converse of disorder predictions, as the overlap between the two sets constitutes 13.8% of the total number of amino acids in the human proteome (Fig. 3A). Similar overlap percentages are observed for other eukarya (10.9% (*S. cerevisiae*), 13.8% (*P. falciparum*), Table 2). For *E. coli* and especially for *A. fuldigus*, the overlaps are much lower (3% and 0.9%, respectively), but represent similar ratios of the total number of amino acids predicted as disordered by IUPRED. We made several additional statistics, especially in order to clarify the structural meaning of these three distinct datasets (H2CD, IUPREDdis and H2CD ∩ IUPREDdis).

**Table 2:** Comparison of SEG-HCA and IUPRED predictions.

	*H. sapiens*	*S. cerevisiae*	*P. falciparum*	*E. coli*	*A. fulgidus*
**Residues in H2CD**	86.3%	90.2%	89.4%	97.8%	99.4%
**Disordered residues (IUPRED-L)**	27.5%	20.7%	24.4%	5.3%	1.5%
**Residues in the H2CD ∩ IUPRED set (overlap)**	13.8%	10.9%	13.8%	3.2%	0.9%
**PDB (relative to the total PDB residues)**					
**H2CD only**	89.7%	91.2%	96.3%	94.6%	98.8%
**H2CD ∩ IUPRED**	6.4%	4.9%	2.2%	3.5%	0.7%
**IUPRED only**	3.9%	3.9%	1.5%	1.9%	0.5%
**PDB-MOBI (relative to the total PDB-MOBI residues)**					
**H2CD only**	92.4%	94.1%	99.8%	95.3%	99.1%
**H2CD ∩ IUPRED**	5.4%	3.9%	0.2%	3.3%	0.6%
**IUPRED only**	2.4%	2%	0.1%	1.4%	0.3%
**SCOP (relative to the total SCOP residues)**					
**H2CD only**	92.1%	94.3%	97.5%	95.2%	98.6%
**H2CD ∩ IUPRED**	5.4%	3.8%	1.7%	3.3%	0.9%
**IUPRED only**	2.4%	1.9%	0.8%	1.5%	0.5%
**ANCHOR (relative to the total residues in the set)**					
**H2CD only**	6.4%	4.4%	7.5%	1.3%	0.3%
**H2CD ∩ IUPRED**	46.1%	44.7%	39.8%	11.1%	9%
**IUPRED only**	32.6%	24.4%	15.5%	10%	1.8%
**ANCHOR (relative to the total ANCHOR residues)**					
**H2CD only**	29.9%	32.7%	44.1%	68.9%	77.2%
**H2CD ∩ IUPRED**	41.2%	45.1%	43.1%	18.9%	20%
**IUPRED only**	28.9%	22.2%	12.8%	12.3%	2.8%
**CD (relative to the total residues in the set)**					
**H2CD only**	50.6%	49.1%	23.4%	72.6%	63.8%
**H2CD ∩ IUPRED**	15.4%	14.1%	3.8%	60.5%	49.2%
**IUPRED only**	8.7%	9.4%	3.5%	40.4%	34.5%
**CD (relative to the total CD residues)**					
**H2CD only**	91.7%	94.1%	95.2%	96.1%	99%
**H2CD ∩ IUPRED**	5.3%	3.7%	2.8%	2.7%	0.7%
**IUPRED only**	3.0%	2.2%	2%	1.2%	0.7%

Percentages of amino acids covered by SEG-HCA and IUPRED-L predictions in the proteomes of *H. sapiens* (18395802 residues), *S. cerevisiae* (2828010 residues), *P. falciparum* (3979079 residues), *E. coli* (1251206 residues) and *A. fulgidus* (655015 residues). Each class (SEG-HCA, IUPRED and SEG-HCA∩IUPRED) has been searched for coverage by 3D structures (PDB, SCOP classes A to F and PDB filtered with MOBI-DB), ANCHOR predictions and CDD assignments.

**Figure 4:**
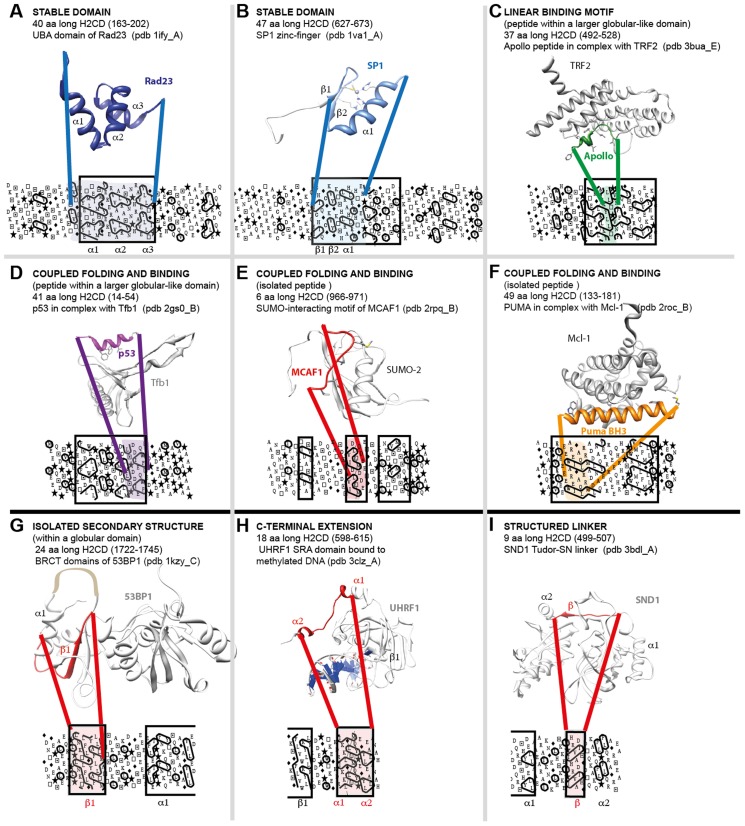
Structural features of small H2CD. Small H2CD represent a large part of the total number of the predicted H2CD (64814 (47%) in the human proteome). In order to understand their structural behavior, we searched for small H2CD (length <50 amino acids), which are covered by CD and show significant sequence similarities with experimental 3D structures. Several cases are illustrated here, from panel A to I. Panels A, D, E, F, H are included in the SEG-HCA∩IUPREDdis set, whereas panels B, C, G and I are found in the “SEG-HCA only” set.

We first evaluated the overall match of protein sequences included in the three datasets with PDB information. Most of the regions (96.1%) covered by PDB (14.4% of the total number of amino acids) are included in the H2CD sets (H2CD and H2CD ∩ IUPREDdis), indicating that most of the 3D structures included in PDB well cover H2CD predictions (Fig. 3B and Table 2). However, PDB files may include some disordered regions. Then, in order to select only regions with defined 3D structures, we filtered the PDB assignments for disorder using MobiDB, a recent comprehensive centralized database on different flavors of disorder [34], including the well-known DisProt database [35]. We also considered classes A to F of the SCOP database [36], covering globular as well as transmembrane domains. Although these procedures are likely not sufficient to completely remove all disordered regions, the results clearly show that amino acids from PDB-filtered files and SCOP A to F classes are well covered by amino acids in H2CD (97.8% and 97.5%, respectively, Fig. 3B). This is confirmed on the different proteomes analyzed here (Table 2). We further examined examples of PDB sequences included into each set. Pure H2CD are found associated with 3D structures of globular or membrane domains, whereas pure IUPREDdis mainly correspond to small segments without regular secondary structures (*e.g.* linker between two domains, pdb 3qp5(A) (aa 312–321), or unstructured interacting peptide, pdb 1e4g(P) (aa881–895)). In contrast, many examples were found In the H2CD ∩ IUPREDdis set, where the binding of a partner is coupled to folding (*e.g.* the alpha helix of the p53 TAD, which folds upon Tfb1 binding (pdb 2gs0(B) [37], Fig. 4D), the CREB KID domain interacting with the KIX domain of CBP (pdb 1kdx(B) [38]) and the BH3 domain from PUMA interacting with Mcl-1 (pdb 2roc(B) [39], Fig. 4F)). The two complementary interaction domains of mouse CBP and human ACTR, which undergo synergistic folding, were also detected in the H2CD ∩ IUPREDdis set (pdb: 1kbh [40]). Some rare examples were also found of small stable globular domains, as illustrated in Fig. 4A. Worth noting is that small sequence segments of the same ET domain structural family [41], also falling in this H2CD ∩ IUPREDdis category, behave either as stable domains (BRD4 ET domain, pdb 2jns(A) [42]) or as IDP undergoing coupled folding and binding (AF9 ET-like domain 2lm0(A) [43]). This indicates that for small domains, the distinction between stable and unstable 3D structures may be tenuous. Of note is that the H2CD ∩ IUPREDdis set principally contains small H2CD (mean length 28 amino acids, versus 159 amino acids for H2CD not covered by IUPREDdis predictions (70% coverage), also see below).

As shown above, known 3D structures in the H2CD ∩ IUPREDdis set include several segments that undergo coupled folding and binding. We thus wondered if the overlap between H2CD and IUPREDdis may actually correspond to regions predominantly predicted by ANCHOR, a predictor of disordered regions that undergo binding transitions during protein-protein interaction, using the same energy estimation than IUPRED [44], [45]. ANCHOR predictions fall in the three sets, but represent the highest ratio in the H2CD-IUPREDdis set (46.1%, versus 32.6% and 6.4% in the IUPREDdis and H2CD sets, respectively, Fig. 3C). This strongly supports that the overlap between H2CD and disordered predictions is enriched in segments that fold on binding and that the comparison between H2CD and IUPREDdis may allow the definition of distinct categories within disorder.

Second, we also estimated the coverage of order predicted by different approaches: a) H2CD prediction, b) IUPRED prediction of order (ordIUPRED), c) converse of IUPRED prediction of disorder (convdisIUPRED) and d) ANCHOR prediction. H2CD predictions well cover the ordIUPRED and convdisIUPRED predictions (95% and 95% coverage, respectively). However, the ordIUPRED and convdisIUPRED predictions cover only partially the H2CD predictions (59% and 63% coverage, respectively), highlighting a larger coverage of order by SEG-HCA predictions, consistently with results presented in Fig. 3A (H2CD-IUPREDdis overlap). 80% of the amino acids highlighted by ANCHOR are covered by H2CD, indicating that ANCHOR predictions include a large proportion of segments with high density in hydrophobic clusters. ANCHOR predictions not covered by H2CD are generally small (mean length 13 amino acids) and have few hydrophobic amino acids (mean 18%). We also calculated the proportion of small H2CD (length ≤50 amino acids) which are covered by ANCHOR predictions and observed that these cover only 60% of H2CD. On average, small H2CD not covered by ANCHOR predictions are 19 amino acids long and possess 29% hydrophobic residues.

A propensity for folding might thus be predicted for H2CD with a strong IUPREDdis signal, as it is the case for the four examples of coupled folding and binding mentioned above or that shown in Fig. 4E. Using this rule, small stable 3D structures, such as the UBA domain of Rad23 (Fig. 4A) may also be picked out. In contrast, regions without any H2CD signal might be then classified as “non foldable” segments. This represents on average 23% of the protein residues in the human proteome, in agreement with previous estimations (21.6% in Ward 2004). Similar trends were observed for *S. cerevisiae* and *P. falciparum*, whereas in *E. coli* and *A. fulgidus*, this percentage is lower (10% and 6%, respectively) (Table 1).

### Comparison with domains assigned from the Conserved Domain Database gives access to orphan sequences

We collected for each protein the CDD assignments (as found using RPS-BLAST [24]) and discarded multi-domains, as these are already counted with domains. 69348, 7251, 5161, 5497 and 2679 CD were identified in the proteomes of *H. sapiens*, *S. cerevisiae*, *P. falciparum E. coli* and *A. fulgidus*, respectively (Table 1).

The number of predicted H2CD is approximately twice higher than the number of CD: 137665 and 18555 for *H. sapiens* and *S. cerevisiae*, respectively, whereas it is nearly similar (6745 and 3075) for *E. coli* and *A. fulgidus*, respectively (Table 1). Interestingly, the number of H2CD (22939) is more than 4 four times higher than the number of CD (5161) for the *P. falciparum* proteome. Most (97%) of the CD amino acids (40% of the total number of amino acids) are included in the H2CD set (human proteome H2CD and H2CD ∩ IUPREDdis, Fig. 3D and Table 2). As regards to the highlighted relationship between H2CD and foldable regions (see above), this indicates that CD mainly include foldable domains. The similarity in hydrophobic cluster composition between the CD and H2CD databases, as well as with the SCOP database (classes A to F) further supports their relationship (Fig. S1).

**Figure 5:**
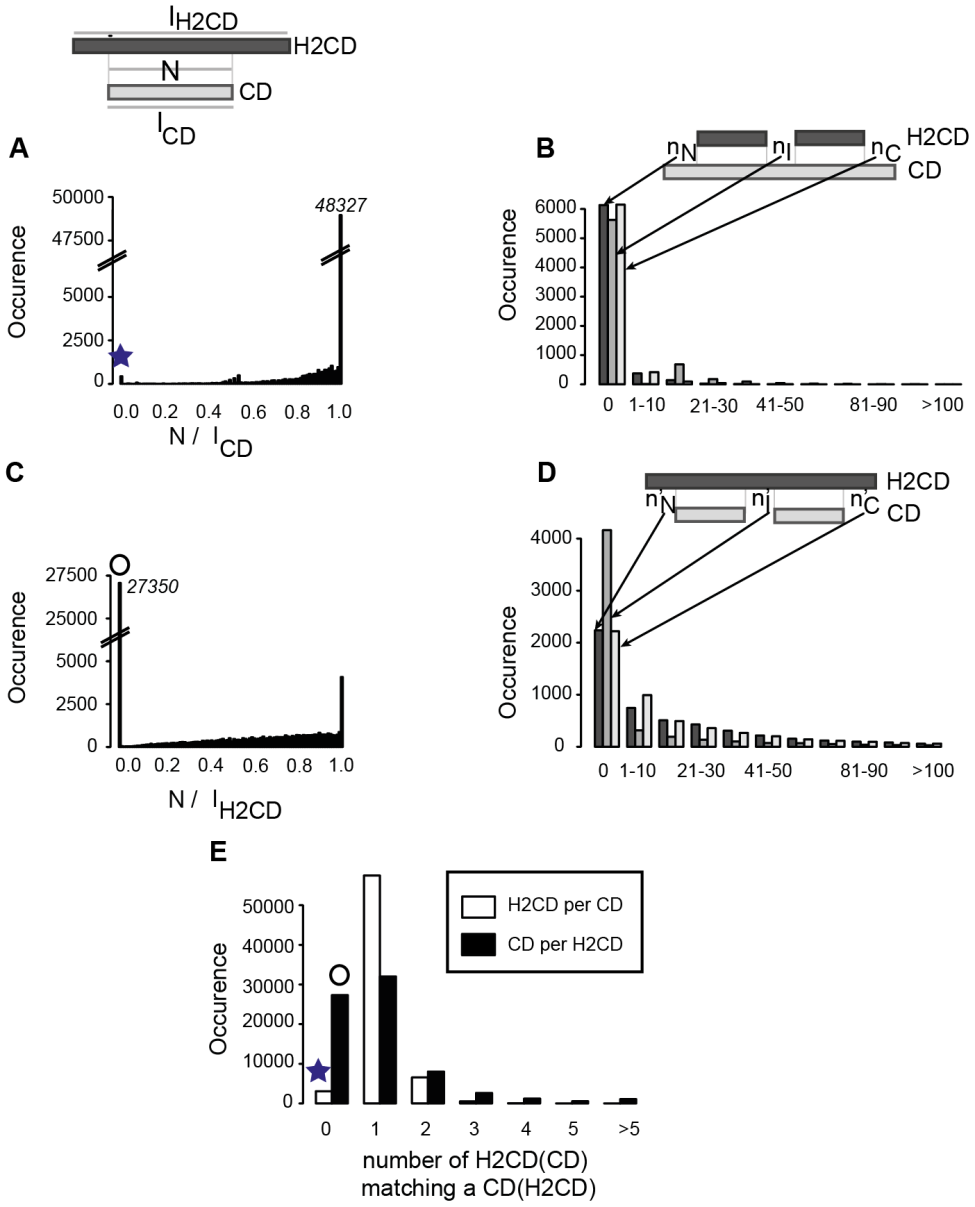
Characterization of segments with high hydrophobic cluster density (H2CD) in the human proteome, relative to CD extracted from CDD. (A) Distribution of the coverage rate of CD by H2CD (N/l_CD_). (C) Distribution of the coverage rate of H2CD by CD (N/l_H2CD_). N is the number of common positions and l_CD_ and l_H2CD_ the total length (in amino acids) of the CD and H2CD, respectively. The star and circle point out CD and H2CD with no or low coverage by H2CD and by CD, respectively. Over-coverage of H2CD by CD and of CD by H2CD are reported in panels (B) and (D), respectively. (E) Number of large H2CD (>50 amino acids) matching a CD (white), number of CD matching a large H2CD (>50 amino acids) (black).

We wondered whether CD, as assigned from CDD, are well detected and covered by H2CD, as predicted by SEG-HCA (Table 1, Fig. 5). First, we calculated the percentages of CD positions, which are predicted as H2CD (Fig. 5A and Table 1). 75% of the CD have up to 95% of their length covered by H2CD in all the proteomes, except *for P. falciparum* and *A. fulgidus*, for which the coverage is even higher (82 and 86%, respectively). These high percentages thus demonstrated that the vast majority of CD are well identified by SEG-HCA, as already observed in Fig. 3D. Only a few number of CD (435 (1,2%) in the *H. sapiens* proteome (star in Fig. 5A and Fig. 3D), and between 0 and 9 in other ones (Table 1)) are not covered by H2CD. These are logically less hydrophobic, with in average 28% of hydrophobic amino acids. Metal ions or disulfide bridges often stabilize some of these domains. Hence, 194 of the *H. sapiens* CD that are not covered by H2CD (45%) correspond to zinc fingers, as deduced from the CDD annotations. We looked more precisely at positions of CD that are not covered by H2CD, either in upstream, downstream or in the middle of CD (Fig. 5B), and observed high peaks for a value of 0, meaning that the limits of CD are well covered by H2CD. The extent of non-coverage appears more pronounced for internal segments of limited length, probably highlighting large loops and/or poorly hydrophobic segments within domains, which are not predicted by SEG-HCA. CD, which don't match any H2CD or match multiple H2CD, can also be highlighted on Fig. 5E and Fig. S2 (white bars).

We also looked at the converse information, *i.e.* the extent to which H2CD are covered by CD. We calculated the percentages of H2CD positions, which are assigned as CD (Fig. 5C). Only 22.4% of the H2CD have up to 95% of their length covered by CD, indicating that CD only partially cover H2CD, as already observed in Fig. 3D. A large number of H2CD have no CD assignment, meaning that they are orphans. They constitute 34% of the H2CD in the human proteome, and up to 72.8% in the *P. falciparum* proteome (72.8%, Table 2). These percentages slightly decreased when considering only large H2CD (>50 amino acids, 19.8% of the total number of H2CD, white circle in Fig. 5C and Fig. 5E (black bar at 0) ; Table 1). These large H2CD have 31% hydrophobic amino acids and an average length of 139 amino acids. These features are very close to those of CD domains (31% hydrophobic amino acids and 111 amino acids long). Only a weak decrease is observed when considering multi-domains, for the definition of CD domains from CDD. The percentage of CD-orphan H2CD range between 8% (*E. coli*) and 22% (*S. cerevisiae*), with the outstanding exception of *P. falciparum*, for which this percentage rises to 54% (Table 1). For this proteome, we further investigated whether amino acid compositions are different within and outside H2CD. As shown in Fig. S3, no clear difference can be highlighted between the *P. falciparum* and *S. cerevisiae* sequences, indicating that the codon usage bias and associated biased amino acid composition affect foldable segments in a similar way than the rest of the protein sequences. Consequently, this biased amino acid composition may explain the resistance of *Plasmodium* H2CD sequences to the detection by CD profiles.

As before, we determined the positions of H2CD, which do not correspond to CD assignments (Fig. 5D), and also observed main peaks for a value of 0, meaning that the limits of H2CD are well covered by CD. Moreover, a higher peak is observed for no mismatch in intermediate positions, likely indicating the preference of 1 CD for 1 H2CD, as also observed in Fig. 5E and Fig. S2 (black bars). However, mismatch occurrences decrease more slowly with the length of the mismatch, likely revealing the importance of partial CD orphans in H2CD.

From Fig. 5E and Fig. S2, it is obvious that most of CD are covered by a single H2CD and vice versa. A small proportion of CD are covered by two distinct H2CD and vice-versa. This can be explained by the fact that SEG-HCA considers either two distinct segments, when large loops or regular secondary structures with few strong hydrophobic amino acids are present within CD (Fig. S4A), or a unique domain when too short or too hydrophobic segments separate two distinct CD (Fig. S4B). Examination of particular cases of non-detected linkers suggests that some improvement of the SEG-HCA prediction tool might be yet expected, by considering for the definition of hydrophobic clusters within potential linkers, alanine residues, which have the highest preference for αlpha-helices.

### Lengths of H2CD segments: Emphasis on small, likely foldable segments, which are not described in CDD

**Figure 6:**
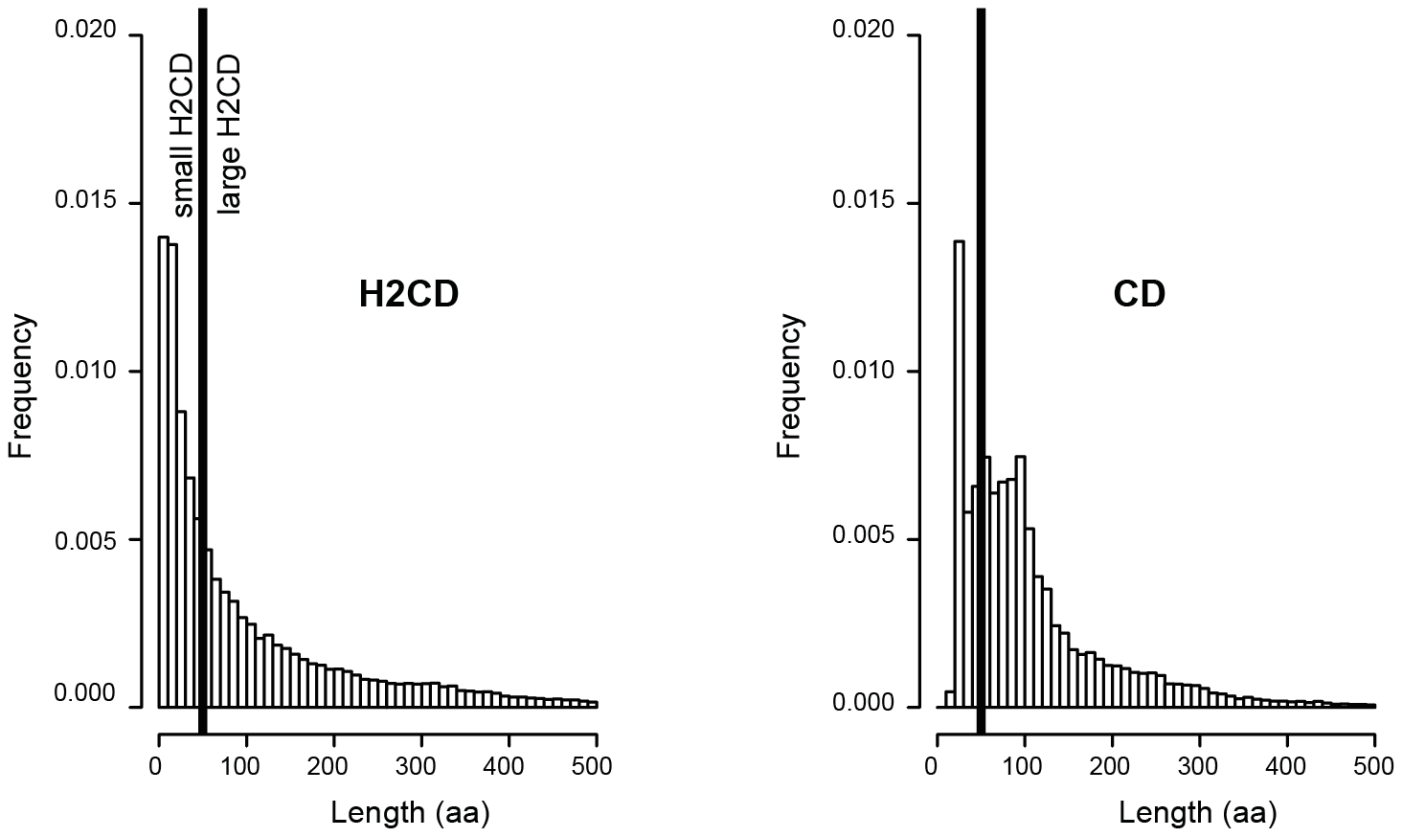
Comparison of H2CD and CD lengths in the human proteome. The frequencies of H2CD and CD extracted from the human proteome are reported as a function of their lengths.

We calculated the distributions of CD and H2CD lengths (Fig. 6 and Fig. S5). Above 50 amino acids, the distributions are quite similar. However, SEG-HCA predicts a lot of small H2CD (length ≤50 amino acids), which are not observed in the CD distribution. Indeed, 64804 (47%), 7823 (42%) and 7591 (33%) of the H2CD from the *H. sapiens*, *S. cerevisiae* and *P. falciparum* proteomes, respectively, have less than 50 amino acids. By comparison, only 27%, 9% and 31% of CD are small-sized. Our results thus also showed that, in three distinct eukaryotic proteomes, it exists a considerable number of short segments, rich in hydrophobic clusters. An in-depth examination of PDB entries matching these segments revealed that they constitute small stable domains (*e.g.* UBA and Zinc-finger domains (Fig. 4A and 4B)), linear interaction motif likely embedded in a small stable domain (Fig. 4C), segments that fold upon contact with partners (Fig. 4D to 4F) or that they are included in larger domains, surrounded by large loops (Fig. 4F), in C-terminal tails (Fig. 4G) and in structured linkers (Fig. 4H). A distinction of segments that may undergo coupled folding and binding could be made by considering overlaps with disorder predictions (see before). The large proportion of small H2CD seems to be specific for eukaryotic genomes. Indeed, in the *E. coli* genome these small H2CD represent 19% of all H2CD and this number decreases to 11% in the *A. fulgidus* genome (Table 1 and Fig. S5). Interestingly, SEG-HCA thus provides a way to access to this information, which is generally missed by standard methods of profile construction (due to their limited size and/or high sequence divergence), as those contributing to CDD.

### CD and H2CD architecture of proteins

**Figure 7:**
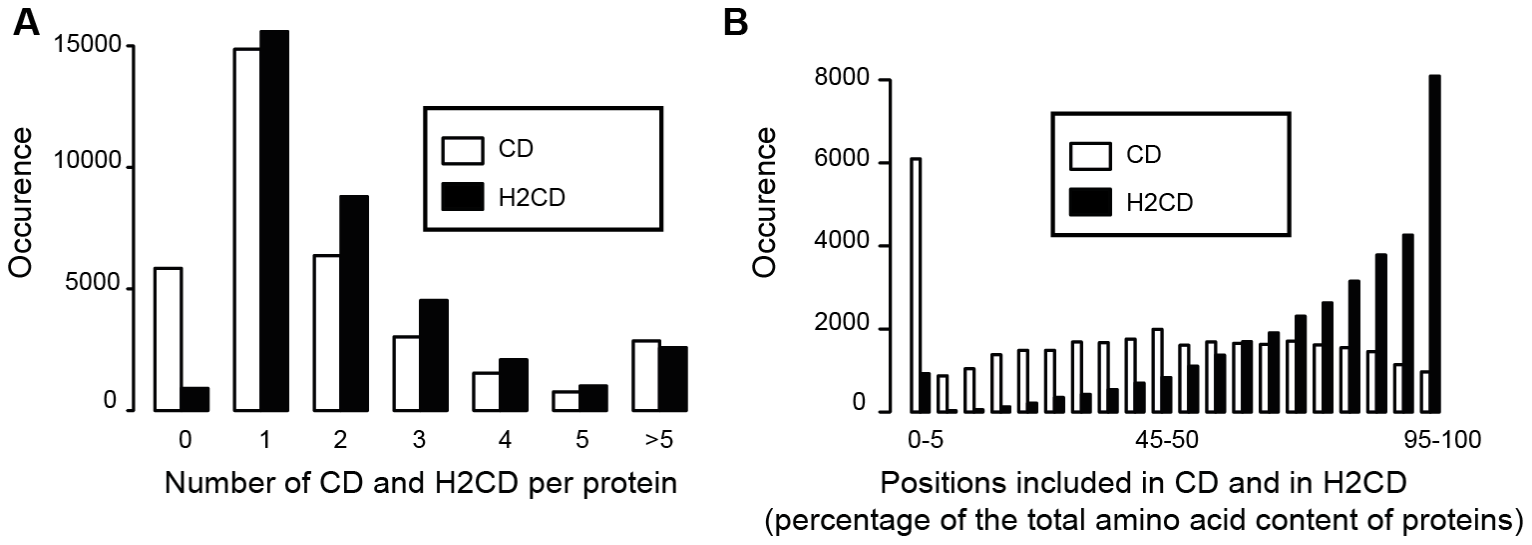
Distribution of H2CD and CD segments among proteins from the human proteome. (A) Number of CD (white) and H2CD (black) assignments per protein. Only H2CD of more than 50 amino acids are considered. (B) Distribution of the number of amino acids (in percentage of the total number of amino acids within a protein) included in CD (white) and H2CD (black) segments.

The percentages of amino acids included in H2CD or CD segments are clearly different (Table 1, Fig. 7A and Fig. S6). On average, 44% of the amino acids of a human protein are included in CD segments, against 77% in H2CD segments. These amino acid coverage values are similar to those found by Ekman and colleagues [2], when considering only the Pfam-A/SCOP assignments or the whole set of predicted domains. The CD amino acid coverage values vary from 29% (*Plasmodium*) to 74% (*E.coli*), whereas elevated H2CD amino acid coverage values are observed in any cases (86% (*Plasmodium*) and 90% (*E.coli*)).

The distributions of CD and H2CD segments within proteins (protein coverage) are also different. Indeed, whereas 17% of the human proteins do not have any CD, only are found 2.6% with no H2CD longer than 50 amino acids (Table 1 and Fig. 7A). This indicates that the number of wholly disordered proteins is very low and that 14% of human proteins contain large foldable domains, which remain uncharacterized (CD-orphan domains). The number of orphan proteins is lower than that reported by Ekman and colleagues, which described that 93% of the eukaryotic proteins could be assigned with a (known or unknown) domain. More generally, the very low percentage of H2CD orphan proteins contrast with previous estimations of fully disordered proteins [46], which are much higher, but these ones include all types of disorder, including regions that are predicted as foldable, and not only natively unfolded segments.

One can also consider the coverage of proteins by only one domain or multiple domains, being advised that one H2CD may cover multiple domains, as discussed from Fig. 5E. Hence, we estimated that the human proteome has 45% and 52% single- and multi-H2CD domain proteins, respectively, whereas bacteria/archaea proteomes have 71%/84% and 26%/14% single- and multi-H2CD domain proteins, respectively (Table 1, Fig. 7A and Fig. S6). This is consistent with the study of Ekman and colleagues [2], which however tipped the balance in favor of multi-domains proteins (65% versus 35% of mono-domain proteins in the human proteome). This last study however fixed the cut-off at 100 residues, and thus did not consider small domains, which are well documented here.

The proportions of proteins with multiple, unique or no H2CD are similar in *Plasmodium falciparum* (56%, 42% and 1%), although the percentages of proteins with multiple and no CD are different (20% and 38% versus 40% and 17% in human proteins) (Table 1 and Fig. S6). This suggests that the higher number of CD orphan sequences in *Plasmodium* sequences may be distantly related to domains existing in other species.

## Discussion

The study presented here provides access to a whole repertoire of foldable H2CD segments in proteomes from the three kingdoms (the eukaryotic genomes of *H. sapiens, S. cerevisiae*, and *P. falciparum*, as well as those of the eubacteria *E. coli* and of the archaea *A. fulgidus*). By a mirror effect, this also gives new insight into disordered regions located outside foldable segments, which are devoid of fold-promoting hydrophobic clusters. Consistently with numerous reports [47], [48], the content in disorder, or precisely non-foldable segments if we consider the converse of SEG-HCA predictions, increases with evolutionary complexity. The major point that our study now highlights is that in eukaryotes, there are many small H2CD segments, whose limited length makes them generally difficult to characterize. This information is in particular absent from or poorly represented in domain databases. Some of these small H2CD segments correspond to isolated stable domains (as exemplified in Fig. 4A and 4B). However, as observed from several case studies (Fig. 4D to 4F), small H2CD segments may also be intrinsically disordered and undergo coupled folding and binding. These segments are generally predicted as IUPs or IDPs by current disorder predictors, and the overlap between disorder and H2CD predictions may thus provide a new interesting way to highlight disorder-to-order transitions, which play key roles for molecular recognition. These segments, which are characterized by structural plasticity and make weak and transient binding, play important roles in regulatory and signaling process [29]. Tools, such as ANCHOR [45] and MorFPred [49], have been developed for detecting such segments, but are based on different principles. MoRFPred is based on a machine learning classifier, based on a comprehensive datasets of MoRFs (Molecular Recognition Features, [49]), whereas ANCHOR relies on pairwise energy estimation, which is also the basis of the disorder predictor IUPred [45]. ANCHOR segments are likely to gain stabilizing energy by interacting with a globular partner. As shown here, ANCHOR predictions are especially found in the overlap between IUPRED (a current disorder predictor) and SEG-HCA predictions. SEG-HCA appears thus well adapted to detect such foldable segments, which have been also named protean segments (ProS, [50]), as it highlights specific features of their interface, enriched in hydrophobic amino acids [45], [51]–[53]. The binding of some foldable segments, such as a beta-strand of MCAF1 (Fig. 4E) and an alpha-helix of BH3 (Fig. 4F), is similar to the folding process and the interface between protein and ligand, richer in hydrophobic residues than the surrounding surface, is similar to the hydrophobic core. In both cases, hydrophobic amino acids participate in the binding interfaces and the hydrophobic cluster has a shape typical of the formed secondary structures. In other situations, such as those presented in Fig. 4C and 4D, the foldable segments are included in small globular-like regions. Hydrophobic amino acids are here likely to participate in both the binding interface and the hydrophobic core of the small globular-like domain in which the peptide is embedded, and the shape of the corresponding hydrophobic clusters deviates from the observed secondary structures [54]. A “folding propensity” may thus be deduced from the consideration of small H2CD, especially included in the H2CD ∩ IUPRED set, provided that these can be distinguished from artifacts (partial domains, as illustrated in Fig. 4G). However, such cases could be solved using evolutionary information.

The residues undergoing coupled folding and binding and participating in the interaction with the partner can usually be mapped in a single continuous segment, and hence, have been connected through common examples to linear motifs [55]. These are collected in the ELM database [56], which captures sequence features shared by common interacting partners. An example is shown here with a peptide of the Apollo (SNM1B) protein (Fig. 4C), a member of the beta-CASP family [57], which form small alpha helices upon binding to the telomere repeat binding factor TRF2 [58], [59]. This peptide is included in a small, 32 amino acid long H2CD. The simplicity of linear motifs offers a good tool for identifying possible partners but generally results in a large amount of false positives. The complementary nature of the two concepts has been recently explored through the comparison of the generic ELM ligand binding motifs (LIG) and ANCHOR predictions [60], indicating that ANCHOR can be used as a structural filter to improve the predictive power of linear motifs. A similar effect or an alternative analysis can be expected from the consideration of the SEG-HCA predictions, which also give information about the structural context of linear motifs. A first calculation made on the ELM LIG motifs showed a mean coverage by H2CD predictions of 67% (with more than a half being predicted as disordered by IUPRED, data not shown). SEG-HCA is however limited to linear motifs including hydrophobic amino acids and thus does not address hydrophilic linear motifs.

Another major observation of our work is that in eukaryotic proteomes, the number of H2CD is approximately higher than the number of domains assigned from the conserved domain database, revealing a lot of CD-orphan domains, which are otherwise not considered by other predictive methods based on homology searches. Orphans domains have either evolved too far from the nearest neighbors to be assigned to a domain or they have been created by some *de novo* mechanisms. Studies have however indicated that most of the solved structures of orphan proteins show structural similarity to already known proteins domains, suggesting that the fraction of orphan domains that have distant homologs is high [61]. A preliminary study on a small set of human CD-orphan H2CD segments reveals that approximately one fifth of them can be assigned by direct inference (from the PSI-BLAST significant results) to already known families of domains (Faure and Callebaut, unpublished data). This study has been performed using the TREMOLO-HCA tool, which combines sequence similarity searches with information on domain architecture and amino acids likely participating in the hydrophobic core [41]. This first emphasizes the sequence divergence of some domain families and the necessity to improve the specificity of associated CD profiles. This also highlights the large amount of putative domains without any known characterized function. These orphan domains are particularly abundant in proteomes from genomes with extreme compositional bias, such as that of the apicomplexan *P. falciparum* (12459 CD-orphan H2CD (81%), Table 1). Previous analyses have already shown the interest of HCA for revealing functional features of such segments [62], which can be now analyzed in a systematic and comprehensive manner.

A recent study [63], [64] has also addressed the problem of regions with no structural domain (SD) assignment (named cryptic domains), through an approach, called DICHOT, for determining structured domains and intrinsically disordered (ID) regions in proteomes. This approach uses sequence conservation in order to distinguish between cryptic structured domains, with no known 3D structures, and disorder. This is fundamentally different from the SEG-HCA approach, which does not use at all sequence conservation for the definition of H2CD segments. Consideration of sequence conservation does not take into account that i) CD domains can have diverged so far that the sequence similarity between family members can be difficult to detect, ii) the sequence of some IDs may share significant similarities with other sequences (this is particularly true for ID sequences undergoing coupled folding and binding). As a consequence, the two approaches are difficult to compare and led to different results, especially for the estimation of the frequency of disordered amino acids, which is much higher in the DICHOT approach (35%, for the human genome).

The comprehensive repertoire described here thus opens new perspectives for the genome-wise characterization of structured domains or potentially foldable regions, as well as for the identification of new domains or motifs, which may play critical functional roles.

## Materials and Methods

### SEG-HCA

SEG-HCA (after SEGmentation through HCA) first identifies the strong hydrophobic amino acids of the sequence, considering for their definition the HCA alphabet (V, I, L, M, F, Y, W) and including cysteine (C) (green in Fig. 2A and B). This alphabet has proven to be optimal, providing the best correspondence between hydrophobic clusters and regular secondary structures [17]. Then, the definition of HCA hydrophobic clusters relies on the consideration of a minimal distance between two hydrophobic amino acids, which is necessary to assign them to separate clusters. This minimal distance, called connectivity distance, is 4 sequential amino acids when the alpha-helix is used as a 2D support for the 2D HCA transposition of the sequence and allows to delineate cluster breakers. These breakers are thus composed of at least four consecutive non-hydrophobic amino acids or a proline (red in Fig. 2A and B). The groups of amino acids between the breakers define hydrophobic clusters, which contain hydrophobic residues (green in Fig. 2A and B), but also may include non-hydrophobic residues (blue-green in Fig. 2A and B), provided the connectivity distance between hydrophobic residues is not reached. As small hydrophobic clusters, containing only one or two hydrophobic amino acids, are not frequently associated with regular secondary structures, these were not considered in our counting if there is no other hydrophobic cluster within their first close neighborhood (7 amino acids) (star in Fig. 2A and 2B). An artificial, simplified binary sequence is then built, where the amino acids composing hydrophobic clusters are represented by 1 and those composing the breakers (which essentially contain non-hydrophobic amino acids) are represented by 0.

From the binary sequence, the percentage of positions included in hydrophobic clusters (HCP, after Hydrophobic Cluster Positions) is computed using an overlapping window of length 17 and assigned to the central position of this window (hexagon in Fig. 2B). This window size was chosen as it corresponds to the segment length encompassing the close neighbors of a central residue on the 2D HCA plot (two rings encircling one central amino acid). This value is similar to the window of 15 amino acids currently used by secondary structure predictors [65]. For the N- and C-terminal eight residues, values were set to those assigned to the ninth and n-8th positions of the sequence, respectively (n being the total length of the considered sequence). The HCP percentages can be plotted, giving a view of the hydrophobic cluster profile of the protein sequence (Fig. 2B). High and low values are associated with high and low densities of hydrophobic clusters, respectively.

SEG-HCA next identifies areas of high hydrophobic cluster density (H2CD), typical of structured or folded regions (Fig. 2B). To that aim, HCP minimal values are identified for a threshold level of 10%, defining thereby potential hinge regions separating these areas of high hydrophobic cluster density (first rough limits, labeled 1 in Fig. 2B). This level is considered as the minimal value, for which and below which a linker is predicted to separate two distinct H2CD. This value was first fixed according to numerous case studies. Optimization of this threshold was considered using a non-redundant (40% sequence identity) SCOP database, downloaded from ASTRAL (http://scop.berkeley.edu/). SEG-HCA was used at different HCP threshold and for each 3D structure, we looked at the values that yield only one H2CD. The distribution of the HCP threshold values was then analyzed, showing an optimum at 22%, thus slightly above 10%. On average, 1.1 H2CD are observed, with 78% coverage. However, it should be noted that the optimization is made on well-stable, already large globular domains. The used threshold is intended to detect such canonical domains, but also small segments, which may be structured and are not (or poorly) represented in the SCOP structural database. Therefore, as supported by several case studies, using the lower threshold of 10% allows the detection of these small segments, in addition to larger ones. At this level, 1.04 H2CD are observed, with 86% coverage (thus values close to those observed for the optimized threshold). Increasing this threshold value may allow the split of multi-domains into domains, but lead to loose small segments, as these last ones generally have low HCP values.

Then, SEG-HCA builds a tree, starting from the observed distances between hydrophobic clusters (leafs). The closest hydrophobic clusters are grouped, constituting nodes, the root (labeled 2 in Fig. 2B), gathering the whole set of hydrophobic clusters contained within the analyzed sequence. SEG-HCA then compares each of the regions identified as the first rough limits (labeled 1 in Fig. 2B) to those defined by the different nodes of the tree. The best overlap between the two is chosen to define the refined limits of the H2CD segment (labeled 3 in Fig. 2B).

SEG-HCA is fully implemented in python v2.7. Scripts are available in Supporting Information (Software S1).

### Datasets

Proteome sequences were downloaded from the National Center for Biological Information (NCBI) (ftp://ftp.ncbi.nlm.nih.gov/genomes/): Hsapiens, Scerevisiae_uid128, Ecoli_042_uid161985, Archeoglobus_fulgidus_DSM_4304_uid57717 and Pfalciparum.

### Comparison with assignments from the Protein Data Bank (PDB), Mobi-DB and SCOP, from the Conserved Domain Database (CDD) and with other predictive tools (IUPRED, ANCHOR)

Each sequence from each proteome was searched for similarities either with the Protein Data Bank (PDB), using the BLASTPGP program version 2.26. Only one iteration was done from each entire protein sequence. Hits sharing more than 95% of sequence identity were selected. The disorder was then filtered by using Mobi-DB ([34], http://mobidb.bio.unipd.it/), which extends the experimental disorder observations found Disprot database to the whole PDB, or by only considering the classes a, b, c, d, e, f from the SCOP structural databases ([36], http://scop.mrc-lmb.cam.ac.uk/scop/). These classes correspond to all alpha protein, all beta protein, all alpha and beta protein (mainly parallel beta sheet), all alpha and beta protein (mainly anti-parallel beta sheet), multi-domain proteins, membrane and cell surface proteins and peptides, respectively.

We obtained information on Conserved Domain(s) (CD) for each protein sequence through the NCBI server (http://www.ncbi.nlm.nih.gov/Structure/cdd/cdd.shtml), which has pre-computed domain architectures. These pre-computed architectures were fixed using the RPBLASTprogram (blast tools version 2.26) with the Conserved Domain Database (version 3.1) [24].

IUPRED was used as a disorder predictor ([30], [31], http://iupred.enzim.hu/). We used the “long” mode (IUPRED-L) to predict disordered segments. A probability to be disordered up to 0.5 was used to consider a position to be disordered. The “glob” mode (IUPRED-G) was used to predict globular domain boundaries from the disordered positions. The “short” mode was not used as it focuses on small disordered regions such as both loops and termini tails.

We used ANCHOR to predict disorder to order transitions ([44], [45] http://anchor.enzim.hu/). ANCHOR is developed from the IUPRED program, and was used with default parameters.

## Supporting Information

Figure S1 **Characterization of segments with high hydrophobic cluster density (H2CD).** Distribution of hydrophobic clusters relative to their length and to the number of hydrophobic amino acids for segments assigned by CDD (A) and predicted by SEG-HCA (H2CD) (B). Hydrophobic clusters whose lengths are greater than 20 amino acids (right part of the figure) are characterized by their percentages in hydrophobic amino acids, rather than by the total number of these amino acids. The two distributions are clearly similar and are typical of that of globular and membrane domains, found the SCOP first classes A to F (C). The longer and more hydrophobic clusters are also present in the three distributions are typical of membrane-spanning domains.
(TIF)

Figure S2 **Characterization of segments with high hydrophobic cluster density (H2CD) in the** ***S. cerevisiae*** **,** ***P. falciparum*** **,** ***E. coli*** **and** ***A. fulgidus*** **proteomes, relative to CD extracted from CDD.** Number of large H2CD (>50 amino acids) matching a CD (white), number of CD matching a large H2CD (>50 amino acids) (black).
(TIF)

Figure S3 **Comparative analysis of the amino acid composition in the** ***P. falciparum*** **and** ***S. cerevisiae*** **proteomes.** The relative frequencies of amino acids of *P. falciparum* versus *S. cerevisiae* proteomes are reported in black. 1 was substracted to each reported value, for improving readability. Positive and negative values are associated with over- and under-representation of the considered amino acid in the P. falciparum proteome, respectively. The four colors are used to highlight the frequencies observed over the whole proteins (black), in regions not included in H2CD (red), within hydrophobic clusters in H2CD (green) and outside hydrophobic clusters in H2CD (blue).
(TIF)

Figure S4 **Correspondence between H2CD and CD.** Examples (**A**) of two predicted H2CD for one CD (large loops (relative to the canonical HCA definition) are present within CD domains) and (**B**) of one predicted H2CD for two distinct CD. In the first case (**A**), a secondary structure lacking strong hydrophobic amino acids (but including alanine residues instead), is considered by SEG-HCA as a potential hinge between two distinct globular-like regions. The same wrong prediction can be observed for very large loops linking two regular secondary structures. In the second situation (**B**), the prediction of a single H2CD is due to a too short or too hydrophobic linker.
(TIF)

Figure S5 **Comparison of H2CD and CD lengths in the proteomes of** ***S. cerevisiae*** **,** ***P. falciparum, E. coli*** **and** ***A. fulgidus*** **.** The frequencies of H2CD and CD extracted from the *S. cerevisiae*, *E. coli* and *A. fulgidus* proteomes are reported as a function of their lengths.
(TIF)

Figure S6 **Distribution of H2CD and CD segments among proteins from the** ***S. cerevisiae*** **,** ***P. falciparum, E. coli*** **and** ***A. fulgidus*** **proteomes.** (Top) Number of CD (white) and H2CD (black) assignments per protein. Only H2CD of more than 50 amino acids are considered. (Bottom) Distribution of the number of amino acids (in percentage of the total number of amino acids within a protein) included in CD (white) and H2CD (black) segments.
(TIF)

Software S1 **SEG-HCA identifies segments with high hydrophobic cluster density (H2CD) as deduced from HCA approach.**
(GZ) Click here for additional data file.
