# Supplementary figures and images for: Comprehensive Repertoire of Foldable Regions within Whole Genomes
Source: PLoS Comput Biol. 2013 Oct 24;9(10):e1003280. doi: 10.1371/journal.pcbi.1003280 (PMC3812050; doi:10.1371/journal.pcbi.1003280)

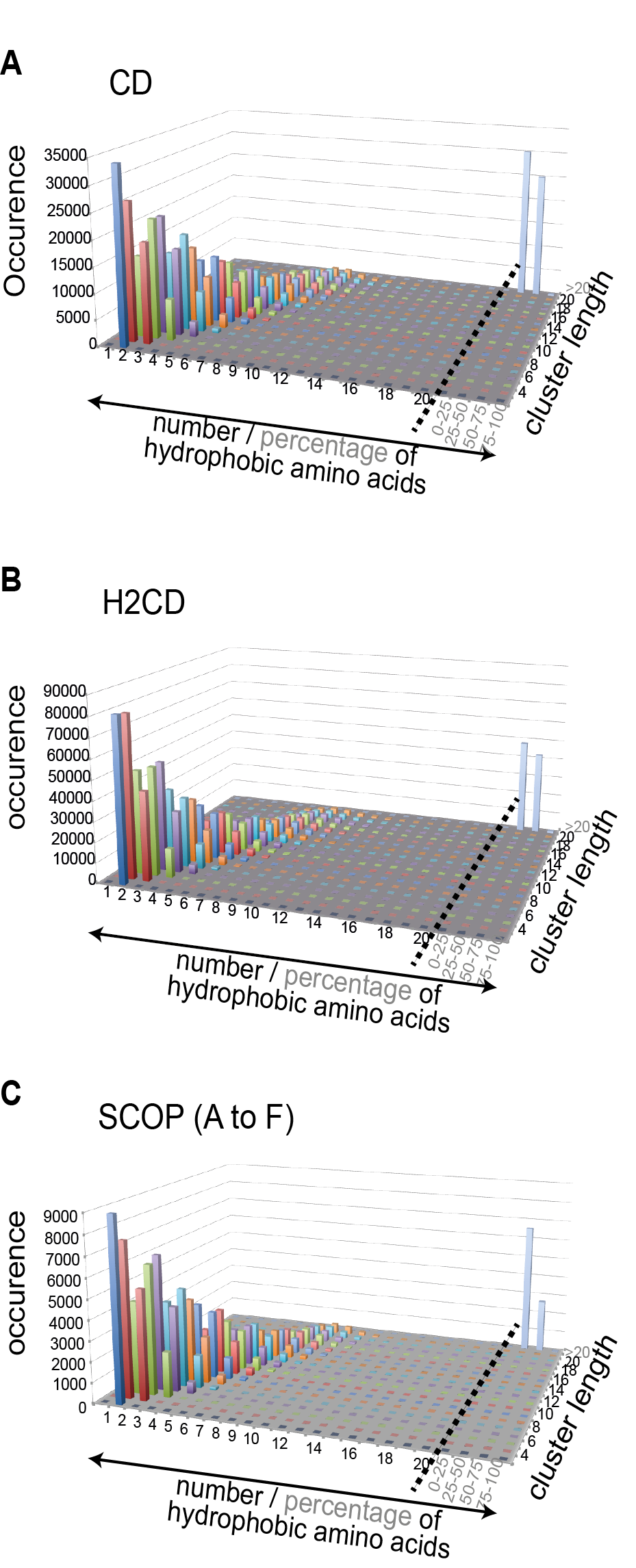

Supplement: Figure S1 — Characterization of segments with high hydrophobic cluster density (H2CD). Distribution of hydrophobic clusters relative to their length and to the number of hydrophobic amino acids for segments assigned by CDD (A) and predicted by SEG-HCA (H2CD) (B). Hydrophobic clusters whose lengths are greater than 20 amino acids (right part of the figure) are characterized by their percentages in hydrophobic amino acids, rather than by the total number of these amino acids. The two distributions are clearly similar and are typical of that of globular and membrane domains, found the SCOP first classes A to F (C). The longer and more hydrophobic clusters are also present in the three distributions are typical of membrane-spanning domains. (TIF) [file pcbi.1003280.s001.tif]

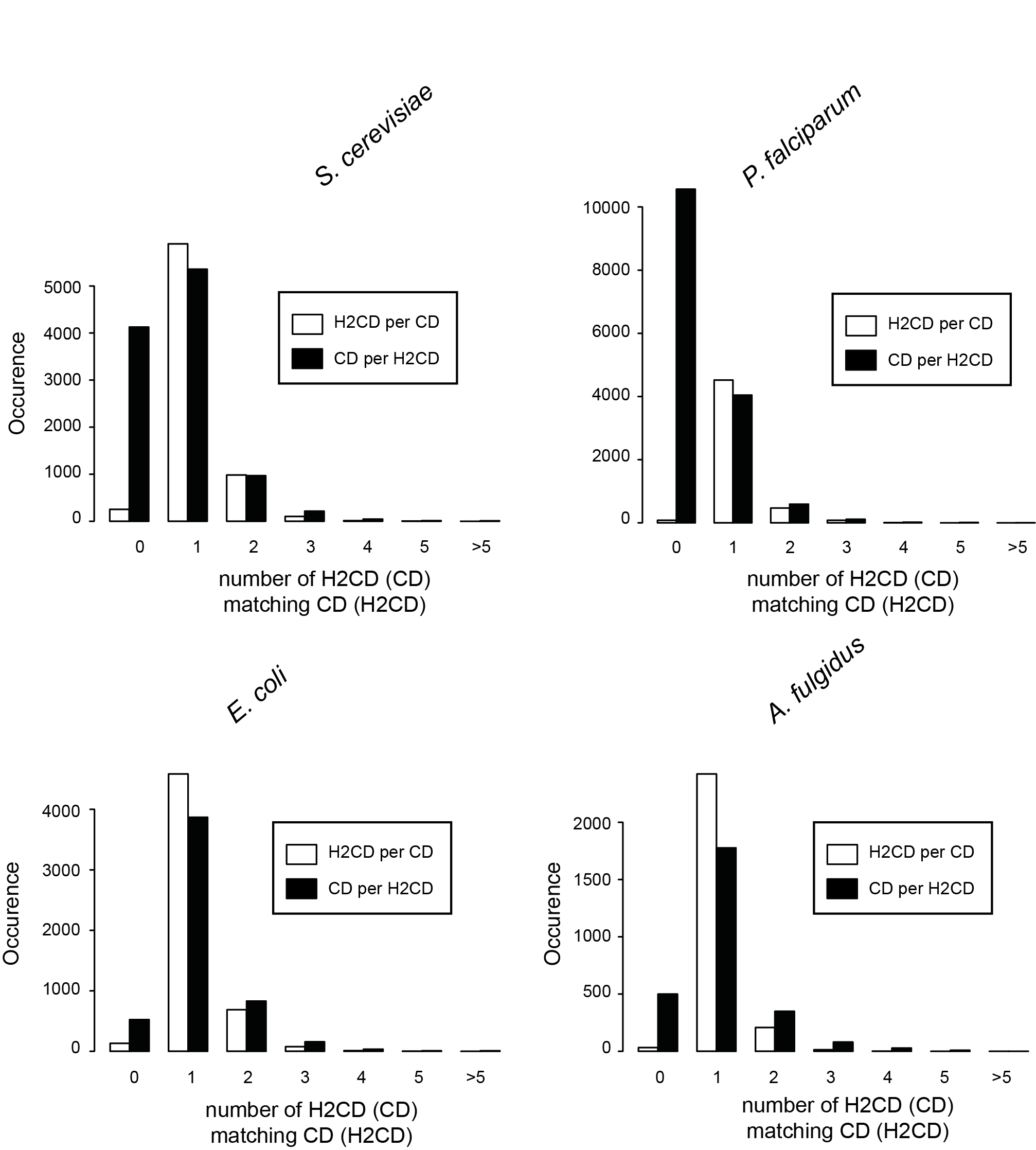

Supplement: Figure S2 — Characterization of segments with high hydrophobic cluster density (H2CD) in the S. cerevisiae , P. falciparum , E. coli and A. fulgidus proteomes, relative to CD extracted from CDD. Number of large H2CD (>50 amino acids) matching a CD (white), number of CD matching a large H2CD (>50 amino acids) (black). (TIF) [file pcbi.1003280.s002.tif]

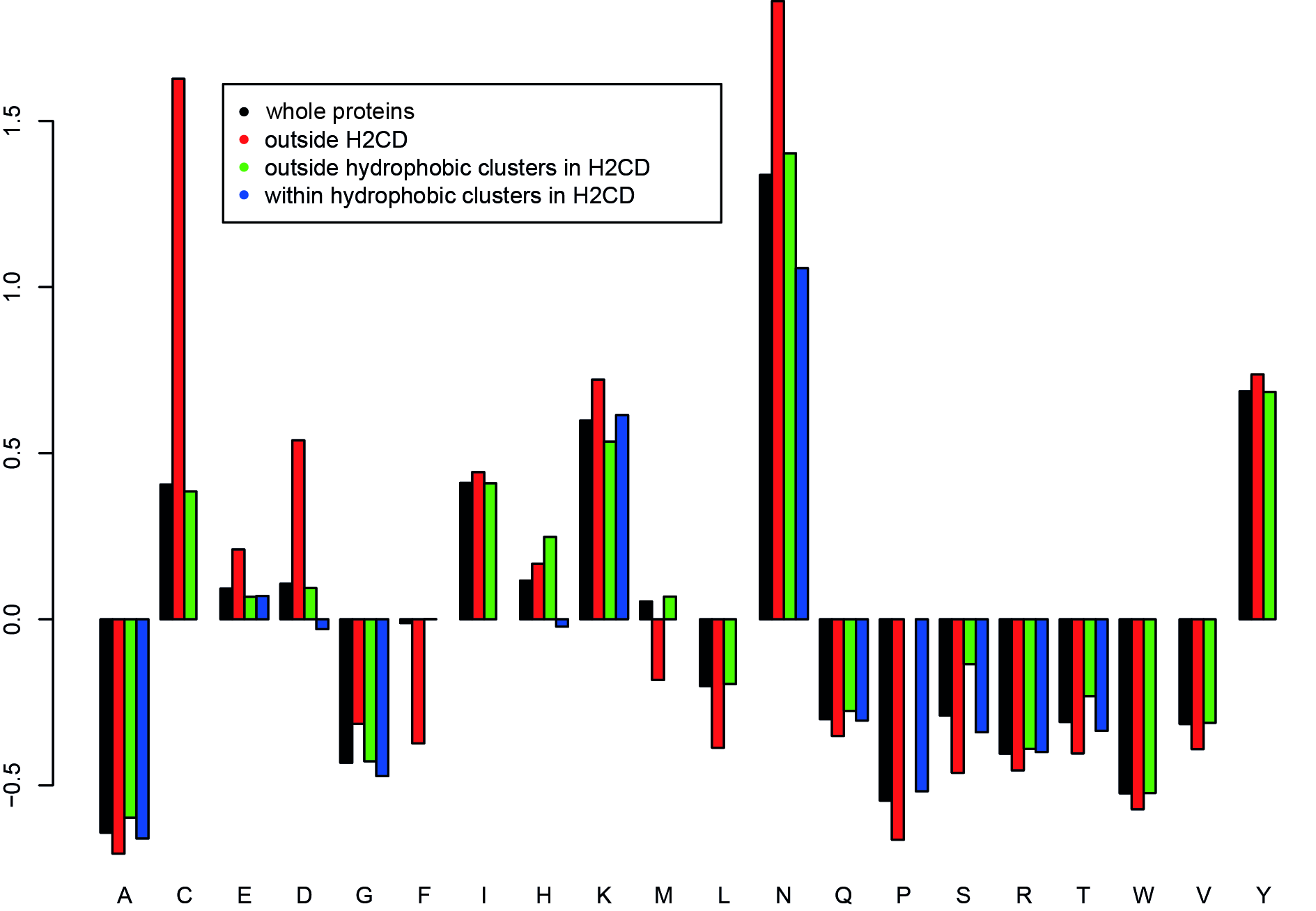

Supplement: Figure S3 — Comparative analysis of the amino acid composition in the P. falciparum and S. cerevisiae proteomes. The relative frequencies of amino acids of P. falciparum versus S. cerevisiae proteomes are reported in black. 1 was substracted to each reported value, for improving readability. Positive and negative values are associated with over- and under-representation of the considered amino acid in the P. falciparum proteome, respectively. The four colors are used to highlight the frequencies observed over the whole proteins (black), in regions not included in H2CD (red), within hydrophobic clusters in H2CD (green) and outside hydrophobic clusters in H2CD (blue). (TIF) [file pcbi.1003280.s003.tif]

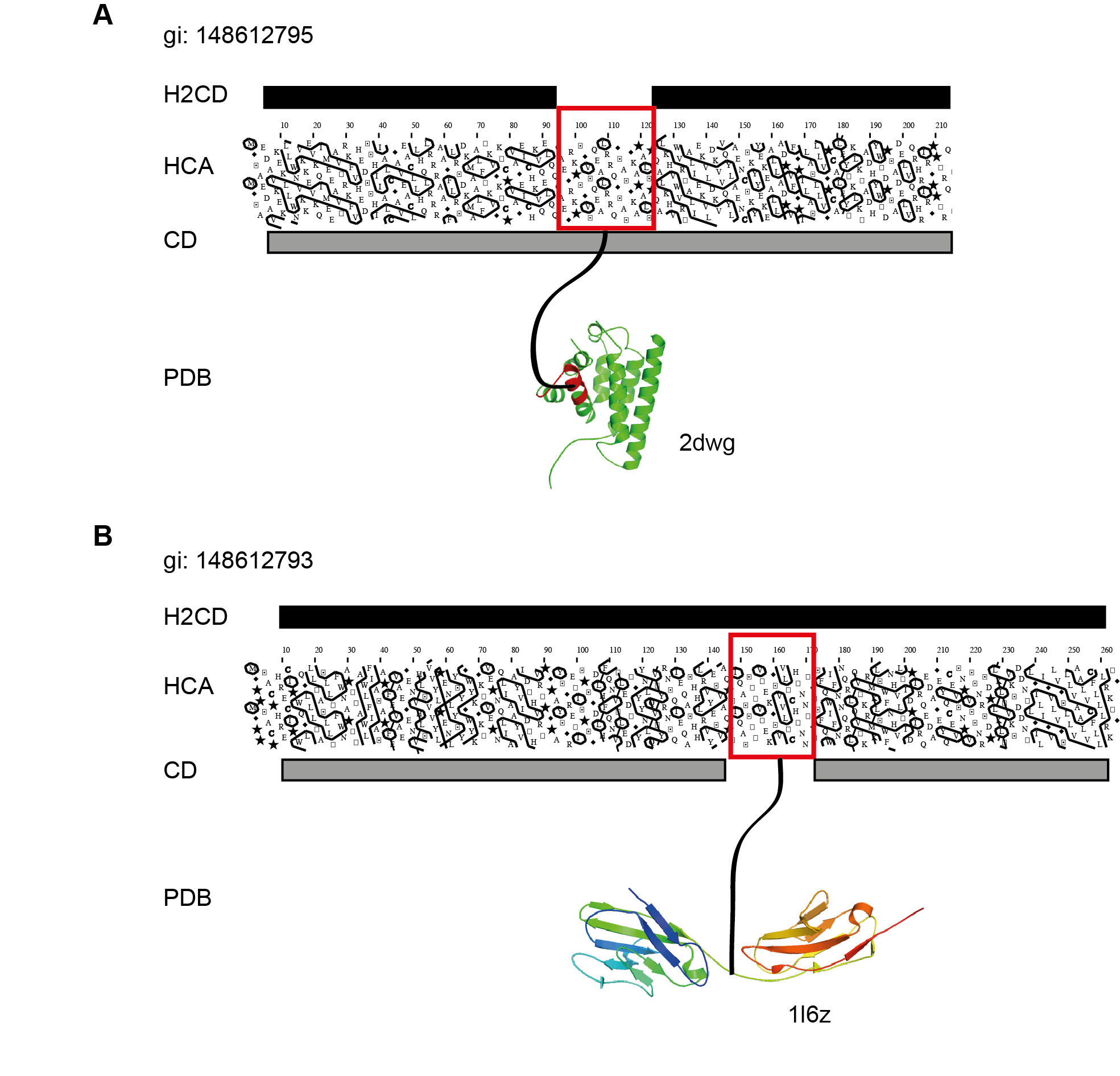

Supplement: Figure S4 — Correspondence between H2CD and CD. Examples (A) of two predicted H2CD for one CD (large loops (relative to the canonical HCA definition) are present within CD domains) and (B) of one predicted H2CD for two distinct CD. In the first case (A), a secondary structure lacking strong hydrophobic amino acids (but including alanine residues instead), is considered by SEG-HCA as a potential hinge between two distinct globular-like regions. The same wrong prediction can be observed for very large loops linking two regular secondary structures. In the second situation (B), the prediction of a single H2CD is due to a too short or too hydrophobic linker. (TIF) [file pcbi.1003280.s004.tif]

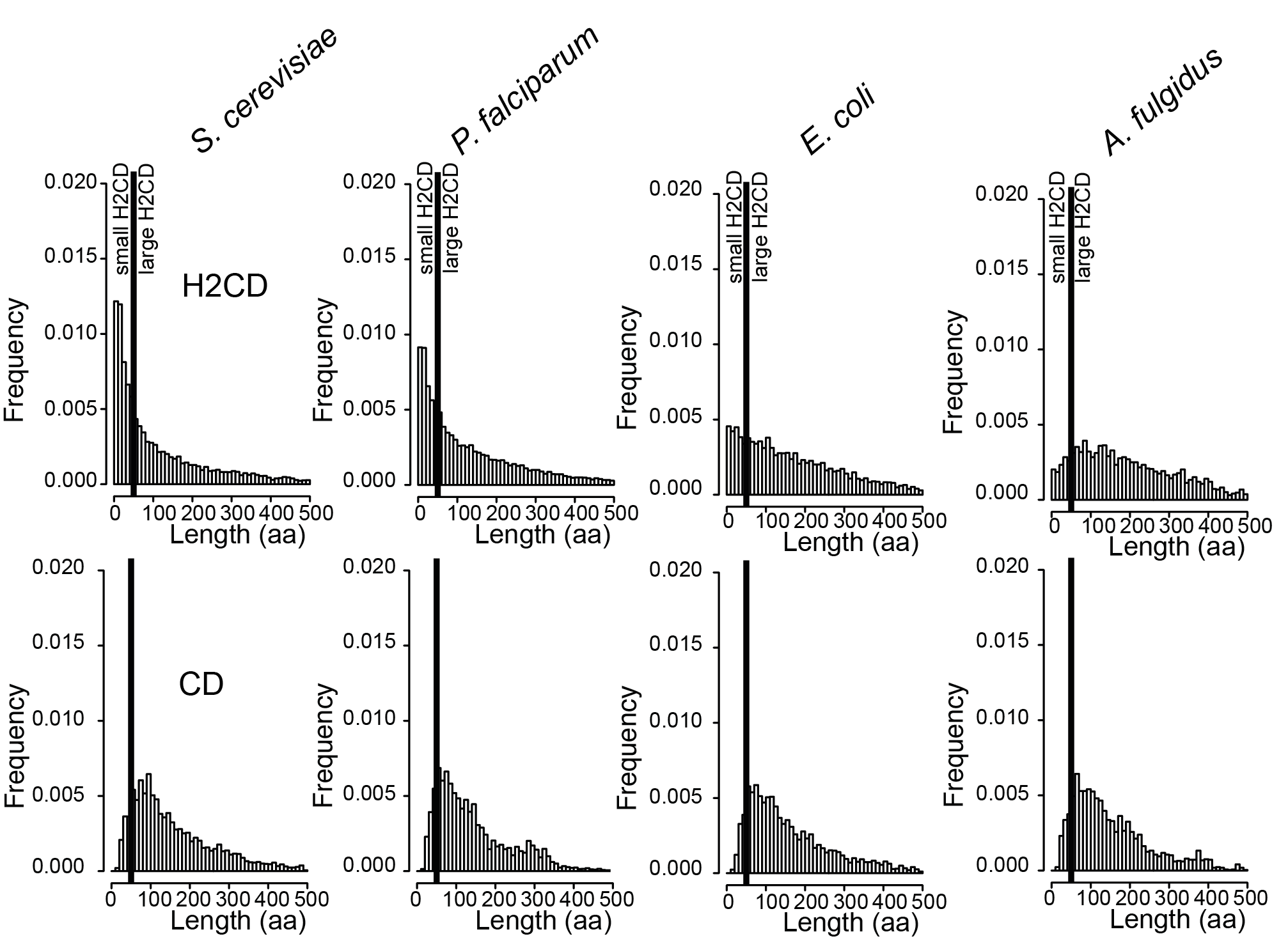

Supplement: Figure S5 — Comparison of H2CD and CD lengths in the proteomes of S. cerevisiae , P. falciparum, E. coli and A. fulgidus . The frequencies of H2CD and CD extracted from the S. cerevisiae, E. coli and A. fulgidus proteomes are reported as a function of their lengths. (TIF) [file pcbi.1003280.s005.tif]

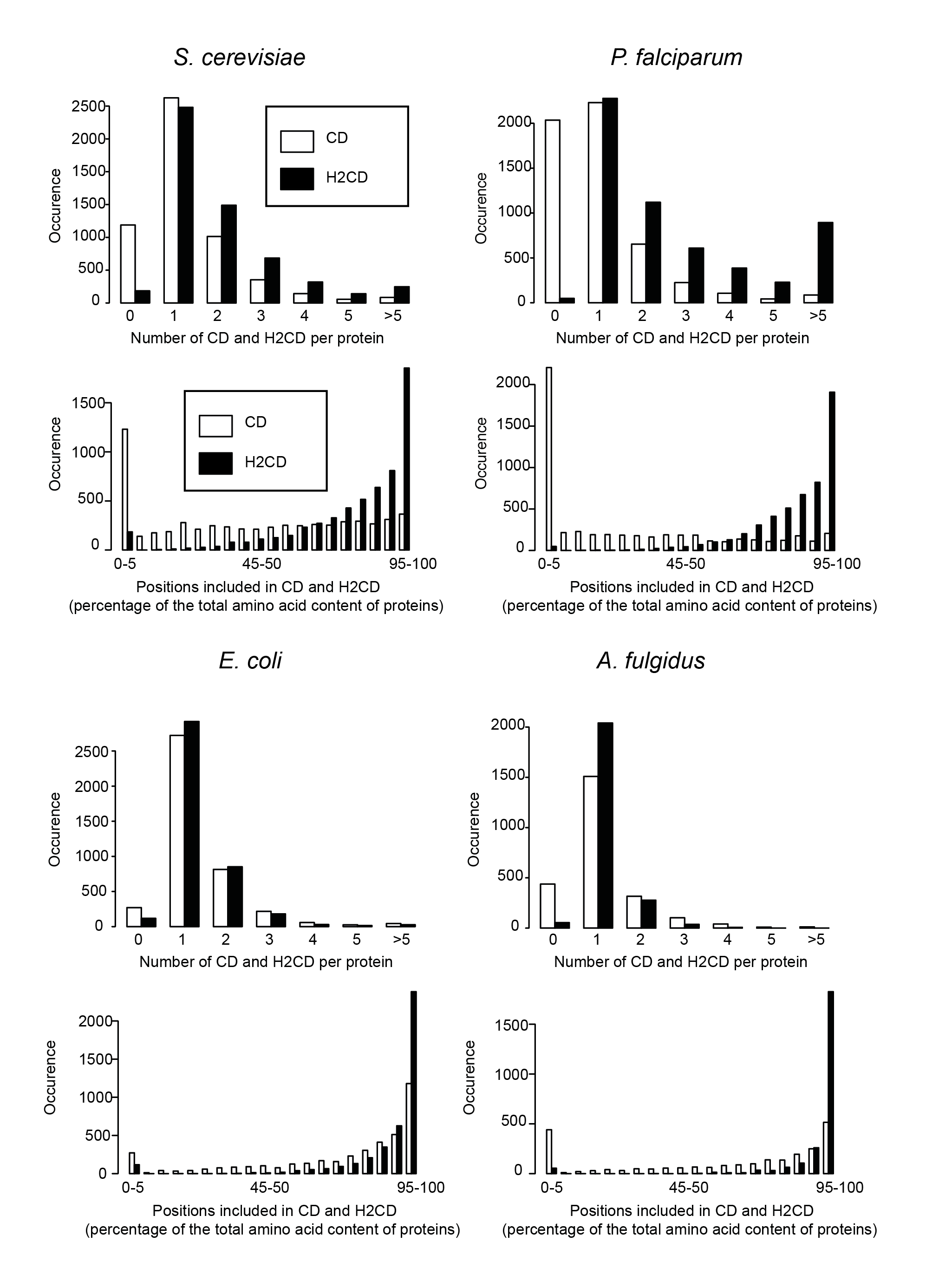

Supplement: Figure S6 — Distribution of H2CD and CD segments among proteins from the S. cerevisiae , P. falciparum, E. coli and A. fulgidus proteomes. (Top) Number of CD (white) and H2CD (black) assignments per protein. Only H2CD of more than 50 amino acids are considered. (Bottom) Distribution of the number of amino acids (in percentage of the total number of amino acids within a protein) included in CD (white) and H2CD (black) segments. (TIF) [file pcbi.1003280.s006.tif]
